# Structure and Aggregation Mechanisms in Amyloids

**DOI:** 10.3390/molecules25051195

**Published:** 2020-03-06

**Authors:** Zaida L. Almeida, Rui M. M. Brito

**Affiliations:** Chemistry Department and Coimbra Chemistry Centre, Faculty of Sciences and Technology, University of Coimbra, 3004-535 Coimbra, Portugal; zalmeida@qui.uc.pt

**Keywords:** misfolding diseases, amyloidosis, oligomers, aggregates, aggregation, aggregation mechanisms, steric zipper, amyloid fibrils, amyloid structure, amyloid dyes

## Abstract

The aggregation of a polypeptide chain into amyloid fibrils and their accumulation and deposition into insoluble plaques and intracellular inclusions is the hallmark of several misfolding diseases known as amyloidoses. Alzheimer′s, Parkinson′s and Huntington’s diseases are some of the approximately 50 amyloid diseases described to date. The identification and characterization of the molecular species critical for amyloid formation and disease development have been the focus of intense scrutiny. Methods such as X-ray and electron diffraction, solid-state nuclear magnetic resonance spectroscopy (ssNMR) and cryo-electron microscopy (cryo-EM) have been extensively used and they have contributed to shed a new light onto the structure of amyloid, revealing a multiplicity of polymorphic structures that generally fit the cross-β amyloid motif. The development of rational therapeutic approaches against these debilitating and increasingly frequent misfolding diseases requires a thorough understanding of the molecular mechanisms underlying the amyloid cascade. Here, we review the current knowledge on amyloid fibril formation for several proteins and peptides from a kinetic and thermodynamic point of view, the structure of the molecular species involved in the amyloidogenic process, and the origin of their cytotoxicity.

## 1. Background

The conversion of normally soluble proteins into insoluble and highly stable amyloid deposits has become the focus of attention by researchers from diverse scientific fields, from physics to chemistry, from biology to medicine. This interest results from the recognition that many of the diseases related with amyloid formation are amongst the most common and debilitating disorders of the modern era [1,2]. Many of these disorders are strongly associated with ageing, such as Alzheimer’s disease (AD) and wild-type TTR amyloidosis (ATTRwt), formerly known as senile systemic amyloidosis (SSA). Alzheimer’s disease affects approximately 50 million people worldwide and it is estimated that this number will exceed 150 million in 2050 [3]. Wild-type TTR amyloidosis affects more than 25% of people over 80 years old, and the disease is becoming more common due to the fast growth of the average age of world population and the increased awareness of medical doctors and positive diagnosis of the disease [4,5]. Presently, there are approximately 50 known amyloid disorders generally referred as protein conformational diseases, misfolding diseases or amyloidosis, with a multitude of distinct symptoms, which are associated with the misfolding and aggregation of several peptides or normally soluble and functional proteins. Table 1 lists some of the amyloid diseases and associated proteins that have been identified to date [1,2]. In the case of local amyloidosis, the amyloid deposits are observed in the organ/tissue where the precursor protein is synthesized; whereas in systemic amyloidosis, the deposition of aggregates and fibrils occurs at locations different than the sites where the precursor protein is expressed [6,7].

As the name suggests, protein misfolding is associated with the formation of an altered protein fold relative to the native structure of a protein. Misfolding can occur as a consequence of several events [8,9,10,11,12,13]: (1) mutations in the gene sequence leading to the production of a protein unable to adopt the native fold; (2) errors in the processes of transcription or translation leading to the production of modified proteins unable to properly fold; (3) failure of the chaperone machinery; (4) mistakes on post-translational modifications or intracellular trafficking of proteins; (5) structural modifications produced by environmental changes; (6) seeding and cross-seeding by pre-formed aggregates or surfaces. Additionally, protein misfolding generally leads to protein aggregation, with specific interactions occurring between intermediate molecular species that tend to form large ordered aggregates, which may evolve into amyloid fibrils which might deposit in the form of insoluble extracellular plaques or intracellular inclusions.

## 2. Protein Aggregation

**Figure 1:**
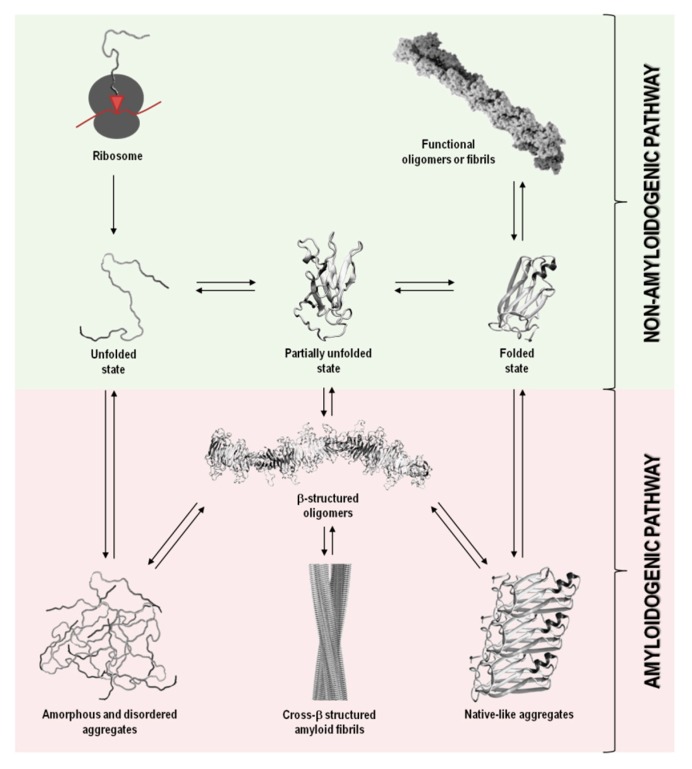
Schematic representation of the variety of conformational states that can be adopted by a polypeptide chain upon biosynthesis in the ribosome. The non-amyloidogenic pathway includes the formation of native sates and functional amyloids. The amyloidogenic pathway associated with pathological states can result from the formation of amorphous aggregates, amyloid aggregates and fibrils, and native-like aggregates. Adapted from reference [1].

In addition to their native structure that intimately correlates with function, proteins also coexist in other states, including disordered, partially unfolded, or multiple aggregation assemblies. Figure 1 summarizes the most relevant protein states, from a mechanistic and biological point of view.

In a functional living system, the multiple conformational states adopted by proteins involve an extremely complex series of thermodynamic equilibria and kinetic barriers, which ultimately are defined by the protein amino acid sequence. Although their intrinsic amino acid sequences and biological environments in which proteins function have co-evolved to preserve proteins in their native and soluble states (non-amyloidogenic pathway), in some conditions, proteins can interconvert into non-functional and cytotoxic protein aggregates (amyloidogenic pathway) (Figure 1) [13,14].

Protein aggregation has been shown to involve natively unfolded or intrinsically disordered systems, such as in amyloid-β (Aβ) peptide in Alzheimer’s disease, or even folded or globular proteins, such as in transthyretin (TTR) associated amyloidoses, where the misfolding process occurs through the formation of partially unfolded states (Table 1 and Figure 1). In most cases, the oligomeric species formed during the amyloidogenic pathway are assemblies of monomeric units. Such aggregates can adopt highly disordered structures, well-defined fibrils with cross-β structure, or also native-like conformations, when originated from unfolded, partially unfolded, or folded monomeric states, respectively (Figure 1). All these types of aggregates have connections with amyloid disorders as they accumulate in well-characterized pathological states and represent a significant manifestation of the multiplicity of mechanisms, structures and morphologies observed during protein aggregation and disease progression (Table 1) [15].

Although the term “amyloid” is often associated with amyloid diseases, the amyloid state can also be present in functional biological processes and contribute to normal cell and tissue physiology (Figure 1). Functional amyloids can be found in bacteria, unicellular eukaryotes, fungi, plants, insects and vertebrates, playing roles as diverse as surface protection and modification, mediation of pathogen-host interactions, pigment biosynthesis, homeostasis control, hormone storage and release, signal transduction, among others [16,17,18,19].

## 3. Amyloid Fibrils

In 1854, Rudolph Virchow found a macroscopic tissue abnormality that exhibited a positive iodine staining reaction, suggesting the presence of starch or amylin like material (from amylum in Latin and amylon in Greek). He then suggested that the abnormal deposits could be cellulose in origin and coined the name “amyloid”. Friedrich and Kekule, in 1859, showed that the deposits were actually protein aggregates, but the designation remained and is still used today [20]. Thus, abnormal accumulations of insoluble highly ordered and fibrillar protein structures in tissues and organs, such as in “misfolding diseases” (Table 1), are known as amyloid fibrils (Figure 1). No sequence, structural or functional correlations are apparent between many of the proteins that display the ability to form amyloid fibrils. The number of amino acids is diverse ranging, for example, from 37 to 44 in Aβ peptide to 691 in lactoferrin (Table 1). Despite these dissimilarities, amyloid fibrils from different protein precursors share many common structural features, namely, the formation of long, unbranched filaments with a cross-β structural motif [21,22,23].

### 3.1. The Tinctorial Properties of Amyloid Fibrils

**Figure 2:**
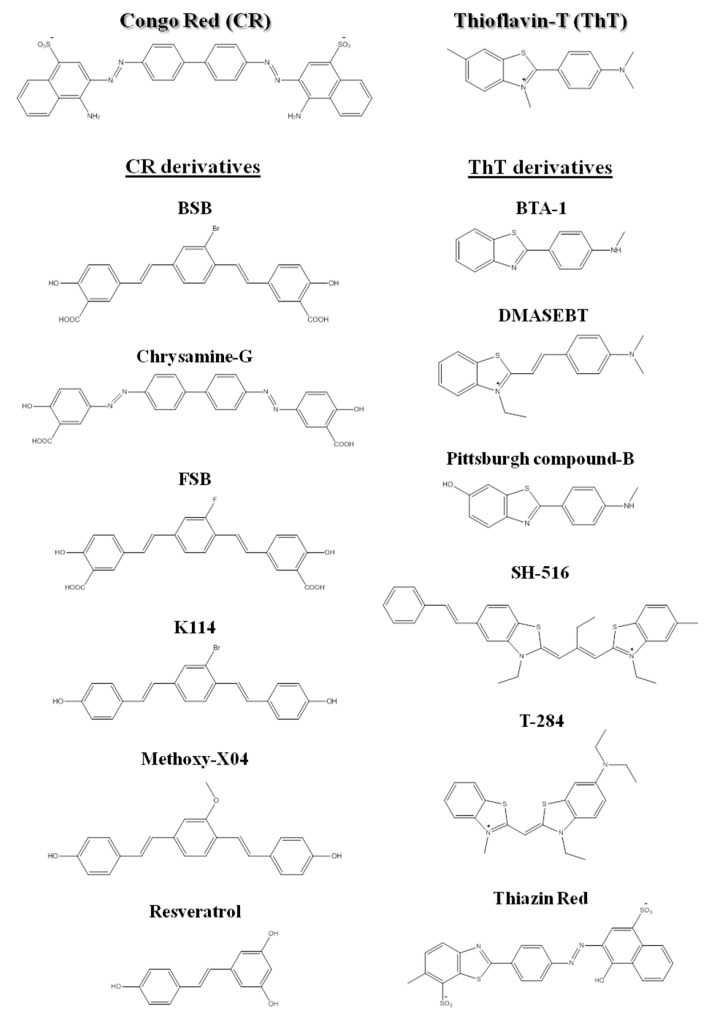
Chemical structures of Congo red, thioflavin-T, and some of their derivatives used in amyloid fibril detection.

From the early days in amyloid research, molecular probes have been used in order to both characterize the mechanisms of amyloid fibril formation, and detect the presence of amyloid in tissues and samples, taking advantage of the distinctive structural features and tinctorial properties of the amyloid material. These molecular probes display changes in spectroscopic properties upon binding to amyloid fibrils. Usually, they are aromatic heterocyclic compounds, some of which toxic [24]. Congo red (CR) and thioflavin-T (ThT), shown in Figure 2, are the most commonly used dyes to identify amyloid material and study amyloid aggregation [25,26,27].

Since the beginning of the 1920s, Congo red has been used for detection of amyloid fibrils. Upon binding to amyloid fibrils, CR displays green birefringence under polarized light, which is associated with a characteristic red shift in the absorbance maximum (from 490 to 512 nm), and presence of a characteristic shoulder peak at approximately 540 nm [28]. CR is the most commonly applied dye in the identification of amyloid in ex vivo tissue slices, using mainly polarization microscopy, but also light microscopy [29] and fluorescence microscopy [30]. However, compared to ThT, CR is less sensitive in the detection of amyloid fibrils [31]. In addition, CR interferes with the aggregation mechanisms and is not appropriate for in situ detection, since it is known to inhibit or to enhance amyloid fibril formation depending on the protein [32,33,34,35,36,37].

More recently, thioflavin-T became a standard dye for amyloid detection showing fluorescence enhancement upon interaction with amyloid deposits in tissue sections [38]. ThT fluorescence emission increases upon binding to amyloid fibrils, with excitation at 450 nm and emission maxima at 482 nm, as opposed to excitation at 385 nm and emission maxima at 445 nm for the free dye [31].

Besides ThT and CR, other compounds have been also used for the detection of amyloid fibrils [39,40,41,42]. The search for novel, more sensitive and blood–brain barrier crossing dyes for detecting the initial stages and more toxic oligomeric species involved in fibril formation in vitro, ex vivo and in vivo is still under way [43,44,45]. Nevertheless, several related structures or derivatives of both CR [46,47,48,49] and ThT [50,51,52,53] have been shown to be quite helpful for both in vitro and ex vivo identification of amyloid fibrils (Figure 2).

The binding mode of the molecular probes to amyloid fibrils is not fully understood. In the case of ThT and CR, a large number of studies examined binding modes and suggested the existence of a binding interface parallel to the long axis of the fibril [54,55].

Features as fibrillar morphology, cross-β structure, and characteristic tinctorial properties are nowadays accepted as benchmarks for defining “amyloid materials”, and any given protein aggregates need to display most of them to be classified as such. In a recent recommendation, the International Society of Amyloidosis (ISA) nomenclature committee suggests the following classification for amyloids based on five main classes, in order to distinguish different natural and synthetic amyloid-like materials: (1) in vivo and ex vivo disease-related fibrils; (2) in vivo and ex vivo functional fibrils; (3) recombinant fibrils of disease-related proteins or functional amyloid proteins; (4) fibrils from synthetic or non-disease-related peptides; and (5) fibrils from hydrogels that produce the cross-β diffraction pattern [56].

### 3.2. Structure of Amyloid Fibrils at the Subunit Level

Since the cross-β motif was identified [57], many studies have been performed to characterize the structure of amyloid fibrils, using different experimental techniques. The combination of all these data has contributed to reveal the structure of amyloid fibrils on a multi-scale basis and to show how individual protein subunits can form cross-β structures.

The structural feature which all amyloid fibrils share is the cross-β motif, characterized by extended β-sheets with individual β-strands arranged in an orientation perpendicularly to the fibril main axis. Amyloid fibrils are unbranched structures with diameters ranging from 2 to 20 nm and usually present several micrometers in length [21,58,59]. The existence of a repeating cross-β structure was first demonstrated in the late 1960s by X-ray fiber diffraction studies [60,61], and later synchrotron X-ray diffraction studies have shown that amyloid fibrils from different amyloid proteins exhibit the same cross-β diffraction pattern [62]. Characteristic amyloid fibril diffraction patterns show a meridional reflection at 4.7–4.8 Å corresponding to the hydrogen bonding distances found between paired carbonyl and amide groups in adjacent β-strands, and an equatorial reflection at 6–11 Å corresponding to the distance observed between stacked β-sheets [62].

**Figure 3:**
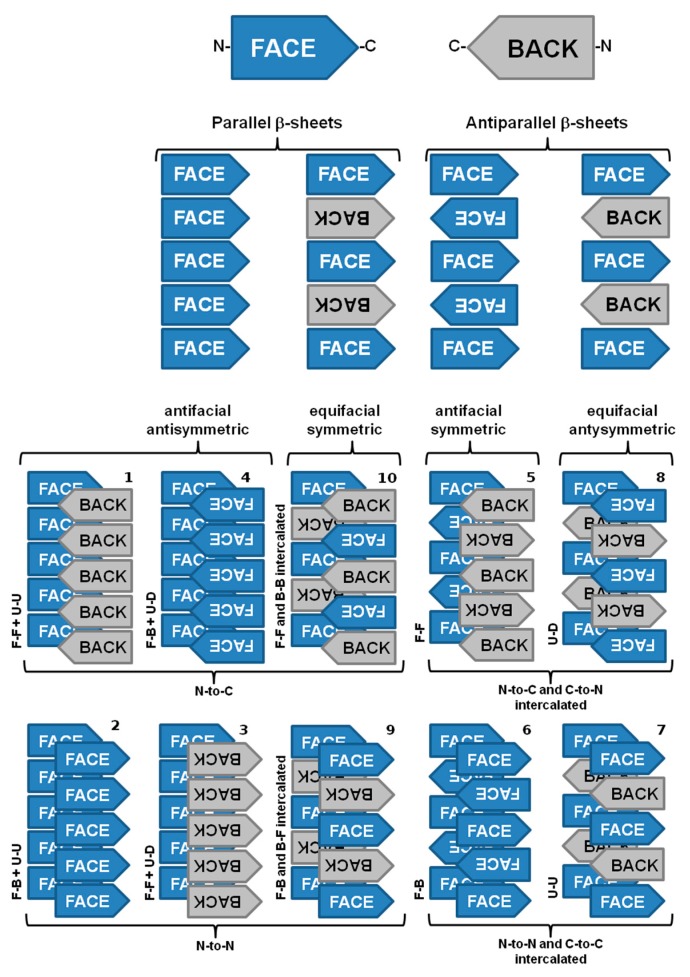
The ten possible amyloid symmetry classes of homo-steric zipper amyloid spines. Legend: face (F), back (B), up (U), down (D), N-terminal and C-terminal. Adapted from reference [65].

Amyloid fibrils from different origins contain a common cross-β spine formed by a pair of β-sheets with the facing side chains of the two sheets interdigitated in a steric zipper [63]. Steric zippers are thus short peptide segments which provide the structural basis for the hierarchical assembly of an amyloid fibril [63,64]. In this sense, stacks of steric zippers are needed to form the cross-β spine of the amyloid protofilament (Figure 3) [65], which constitutes the basic unit of the mature fibril, whereas the rest of the polypeptide chain assumes either native-like or random coil conformation in a peripheral position to the spine [63,66].

Nevertheless, significant variations may be found among the structural arrangement of steric zippers. Assembly of the strands in a sheet produces four patterns of sheet symmetry (parallel versus antiparallel, and antifacial versus equifacial). These four β-sheet patterns are each capable of self-pairing to form cross-β spines. Thus, in total, ten distinct symmetry classes of steric zippers can be enumerated. These classes are distinguishable by the particular sheet pattern and by the symmetry operation that relates the pair of sheets (Figure 3) [65].

Mature fibrils also reveal a periodic structure due to the twist of multiple protofilaments around each other. The helical twist of the protofilaments can give rise to a discernible overall helicity of the mature amyloid fibril. Although the basic structural arrangement of the cross-β structure is conserved in different fibrils, there are different possibilities of how protofilaments can pack into the three-dimensional fibril structure, giving rise to several distinct amyloid fibril morphologies. It is possible to distinguish amyloid fibril polymorphism based on the degree of fibril twisting, the number of protofilaments per fibril, and the diameter or weight per length of the fibrils [22,67,68]. In addition to amino acid composition, the fibril morphology is determined by environmental factors, such as pH, temperature, mechanic agitation, shear force, ionic strength or presence of other co-solutes and co-solvents [22,69].

Amyloidogenic regions or short segments forming steric zipper spines in amyloid fibrils are possible to identify/predict by submitting a protein sequence to specific algorithms and web services. Table 2 summarizes the computational tools available online and reported in the literature to predict propensities or incidence levels of a given amino acid sequence to form amyloid fibrils.

### 3.3. The Cross-α Amyloid-Like Fibril

Cross-β motifs and amyloid fibrils can be formed both by β-sheet rich and α-helical proteins. Alpha-helical conformations have also been associated to amyloid fibril formation. Certain peptides and proteins that present well defined α-helical structures undergo conformational changes into β-pleated structures that in turn aggregate into amyloid fibrils [103,104,105,106,107], while other peptides or proteins maintain its α-helical conformation during the aggregation cascade, forming “cross-α” structures. Cross-α structures are also formed by elongated unbranched fibrils, and display the ability to bind thioflavin-T with the characteristic enhanced fluorescence emission. These fibrils result from the stacking of α-helices perpendicular to the fibril axis [108]. The cross-α structure, although rare, was found in the functional amyloid phenol soluble modulin α3 (PSMα3) [108], in the yeast prion Ure2p [109], in de novo-designed amphiphilic peptides [110,111,112], and among proteins of multiple tandem copies of a helix–loop–helix unit [113].

### 3.4. Hetero-Amyloid Fibrils

The terms “hetero-amyloid” or “hetero-fibrils” relate to amyloid fibrils structures formed by two different β-sheets along a compact hydrophobic interface, featuring an unusual ladder of sequential stacking. The hetero-fibril is stabilized by hydrophobic packing and enriched with interactions along the fibrillar axis, such as hydrogen bonds between neighboring β-strands, analogous to homo-amyloids. This type of amyloid structure was found in the interaction between the M45 protein with other proteins, namely the receptor-interacting protein kinase 1 and 3 (RIPK1 and RIPK3), and the Z-DNA binding protein-1 (ZBP1) [114]; as well as in the RIPK1-RIPK3 necrosome human signaling complex [115].

In addition, it has also been observed that some amyloidogenic proteins may support cross-seeding by amyloid aggregates of different proteins, building a link between different amyloid forms. Heterologous cross-seeding has been reported between amyloid-β peptide (Aβ) and islet amyloid polypeptide [116,117], Aβ and prion protein (PrP) [118], and Aβ and α-synuclein [119]. Furthermore, Aβ fibrils also induce the formation of tau neurofibrillary tangles in vivo [120], and Aβ aggregates promote tau aggregation in vitro [121]. Heterologous cross-seeding was also found when tau K18 interacts with the C-terminus of α-synuclein [122], when functional amyloids curli and Sup35 induce amyloid protein A amyloidosis [123], when PrPs promote Sup35 aggregation [124], and when the in vitro aggregation of CsgA is seeded by CsgB fibrils [125]. Moreover, the observation of heterologous cross-seeding cases suggests that interactions between different amyloidogenic proteins might enhance the onset of certain types of amyloidoses.

### 3.5. The Major Differences between the Techniques that Inform on Amyloid Structure

Recent progresses in instrumentation have improved quite significantly the ability to characterize the structure of complex biological molecules and macromolecular complexes. The knowledge of the three-dimensional structure of amyloid fibrils from different origins provides important insights into the mechanisms of amyloid formation, and thereby helps in the rational design of novel therapeutic agents. The combination of structural biology data from several approaches and experimental techniques provides new insights on how individual protein subunits form fibrillar structures, at an atomic level of resolution. In the past decades, many structural biology studies have been performed based on X-ray fiber diffraction [126], X-ray crystallography [23], solid state NMR (ssNMR) [127], and cryo-electron microscopy (cryo-EM) [128], to mention just a few. Table 3 reports the main differences between these powerful techniques. However, is important to mention that no method is completely effective by itself, since all of them offer unique advantages and limitations.

## 4. Toxic Species in Amyloid Diseases

The pathogenic or the most toxic species in amyloidosis can result from extracellular amyloid fibrillar deposits affecting organ integrity [134,135,136,137], as in the case of cardiac amyloid in transthyretin amyloidosis, and/or soluble protein oligomers that can either populate during the process of amyloid fibril formation or be released by mature deposits causing direct cellular dysfunction [137], as in the case of several neurodegenerative amyloid diseases.

Although the β-sheet structure present in amyloid oligomers is a favorable structural component, it is not an important prerequisite for toxicity [138]. The structural determinants by which these misfolded oligomers cause cellular damage may be related with the exposure of hydrophobic groups on the oligomer surface [139,140] and with the small size of the oligomers with high diffusion coefficient [140,141]. Thus, the toxicity of amyloidogenic oligomers is likely to be a result of their intrinsic misfolded nature and aggregation propensity [137,142]. Such structural properties will cause them to engage in a multitude of abnormal interactions with a range of cellular components, such as phospholipid bilayers, soluble peptides and proteins, protein receptors, RNAs, and cellular metabolites [139,143,144,145,146,147] where some or all of them have the potential to cause cell damage and ultimately cell death. In fact, it is known that several amyloid neurodegenerative diseases have an important inflammatory component which may be the result of the natural response against the “unknown” molecular species formed during the amyloidogenic cascade or against by-products of their action.

## 5. Kinetics and Thermodynamics of Amyloid Fibril Formation

**Figure 4:**
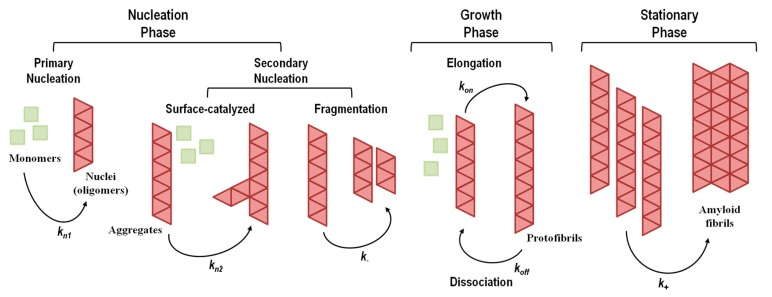
Representative general model for amyloid fibril formation by nucleation-dependent mechanisms (including primary and secondary nucleation) and nucleation-independent mechanisms (absence of nucleation). *k_n1_*, *k_n2_*, *k_−_*, *k_on_*, *k_off_*, and *k_+_* represent rate constants. The stationary phase involves the assembly of protofibrils into mature amyloid fibrils with different morphological structures and a high level of polymorphism. Adapted from reference [148].

The aggregation mechanisms in amyloid systems are prone to multiple pathways, depending on the ensemble of co-existing amyloidogenic conformations and environmental factors. Thus, several aggregation mechanisms and multiple pathways have been described depending on protein sequence, conformational states adopted by the amyloidogenic monomer and experimental conditions (for instance, temperature, pH, protein concentration, and solvent effects). The aggregation processes take place over a wide time range, spanning several orders of magnitude, with conformational changes occurring in the milliseconds and formation of particles observable with the naked eye in days, weeks or months. Elucidation of the mechanisms of amyloid formation and characterization of the most relevant molecular species involved are crucial to devise new rational therapeutic strategies against amyloid diseases. A brief overview of the most common amyloid formation mechanisms is presented in Figure 4, and a summary of the events and conditions that may trigger protein aggregation is shown in Table 4.

The aggregation kinetics of amyloidogenic proteins is highly dependent on protein concentration, and may reflect a nucleation-dependent or a nucleation-independent process (Figure 4) [149,150,151,152].

### 5.1. Aggregation Via a Nucleation-Dependent Mechanism

The nucleation-dependent mechanism of amyloid formation (Figure 4), also known as nucleation–elongation polymerization, displays a typical sigmoidal shape curve as a function of time and consists of three consecutive steps: (1) initial lag or nucleation phase; (2) elongation, growth, polymerization, or fibrillation phase; (3) equilibrium, stationary, or saturation phase [211,212].

The nucleation phase corresponds to the assembly of transient, critical nuclei that next will act as seeding intermediates where additional monomeric subunits can latch on, driving the assembly of oligomers with cross-β structure. At this stage, the rate constants for monomer addition and dissociation are similar, making the global process of nucleation slow and the rate limiting step in fibril formation. The nucleation phase can be shortened or eliminated by the addition of pre-formed aggregates or fibrillar species, a process known as seeding [213,214,215]. In the elongation phase, monomers, nucleus and oligomers continue to interact, assembling into prefibrillar structures that rapidly grow to form ordered fibrillar structures known as protofibrils. Because this phase gives rise to more stable protofibrils, this is a faster and thermodynamically favorable process. Lastly, the saturation phase, where monomer concentration is low and approximately constant, involves the assembly of protofibrils into mature amyloid fibrils with different morphological structures and different levels of polymorphism.

**Scheme 1:**
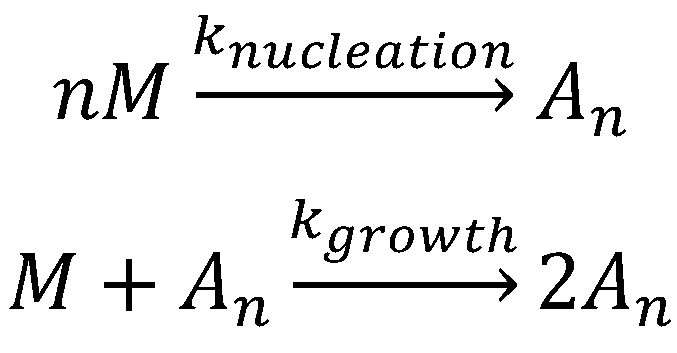
The minimalistic Finke–Watzky mechanism for protein aggregation via a nucleation-dependent pathway, where *M* is the monomer, and *A* is the transient nucleus aggregate. The rate constants for the nucleation and growth steps are *k_nucleation_* and *k_growth_*, respectively [216,217,218].

The Finke–Watzky aggregation model (Scheme 1) is one of the numerous models proposed for nucleation–elongation polymerization and has been applied to more than 40 different aggregating proteins [216,217]. As shown in Scheme 1, the Finke–Watzky model consists of two simple steps: (1) nucleation and (2) growth. Due to its simplicity, this model does have some limitations, including: (1) a vast number of aggregation steps is condensed into two elementary steps; (2) the rate constants, *k_nucleation_* and *k_growth_*, are average rate constants and independent of the size of aggregating species; (3) a higher kinetic order in [*M*] may be kinetically hidden in the nucleation step in particular; (4) all growing polydisperse aggregates are hidden behind the descriptor “*A_n_*”; (5) the descriptor “*A_n_*” can also hide processes such as fragmentation. However, the simplicity and quality of the fits obtained in many practical examples suggest that the Finke–Watzky two-step mechanism encompasses the main characteristics and it is a good general kinetic model for nucleation-growth aggregation.

#### 5.1.1. Primary Nucleation Mechanisms

In the amyloidogenesis cascade, primary nucleation is a critical step in a variety of oligomerization mechanisms and includes the initial formation of amyloidogenic nuclei without contributions from pre-formed oligomers. Two types of primary nucleation mechanisms have been described: homogeneous and heterogeneous. Homogeneous primary nucleation comprises monomers aggregation in bulk solution, while heterogeneous primary nucleation involves association of monomeric subunits on the surface of a different object, such as the wall of a reaction container [191,192,193,194], other proteins [202], phospholipid bilayers [203,204,205,206,207,208,209,210], or the air–water interface [175,176,177,178,179,180].

From a structural point of view, the simplest manifestation of a primary nucleation mechanism is the nucleated polymerization (NP) mechanism. In this case, amyloidogenic monomers aggregate and originate the nucleus, which further grows into amyloid protofilaments and protofibrils through an elongation process involving mostly monomer addition [219,220,221]. This is the preferential mechanism at relatively low protein concentrations favoring the presence of monomeric species in solution.

However, in several instances, it has been observed the presence of multiple conformational heterogeneous oligomers and transient intermediate species during fibril formation which NP mechanisms cannot explain. In these cases, a nucleated conformational conversion (NCC) mechanism has been proposed. NCC comprises structurally organized oligomers as intermediates which are able to subsequently conformationally transition into cross-β dominated fibrillar species. The formation of these conformationally dynamic oligomers may be favored at higher protein concentrations and they undergo a rate-limiting conformational change to form protofibrils and then amyloid fibrils [222]. This type of nucleation was observed, in particular, in the yeast prion protein (Sup35) [223], among variants of the amyloid-β peptide [224,225,226], SH3 domain [227,228,229], Ure2p yeast prion [230], polyglutamine (polyGln) peptides [231], and lysozyme [232,233].

#### 5.1.2. Secondary Nucleation Mechanisms

Although conceptually appealing and observed in several instances, a simple homogeneous primary nucleation mechanism is not always observed [149,223,234]. Several studies have pointed out that simple homogeneous nucleation could not fit certain experimental aggregation kinetics data [235,236]. Simple homogeneous primary nucleation does not take into account other nucleation mechanisms and events, such as fibril-catalyzed secondary nucleation (a monomer-dependent process) and fibril fragmentation (a monomer-independent process) (Figure 4), both contributing to the formation of new aggregation nuclei [237,238,239,240,241]. In fibril-catalyzed secondary nucleation nucleus formation occurs on the surface of an already existing oligomer (Figure 4). No foreign surface is involved in this type of nucleation as in the case of heterogeneous primary nucleation. This nucleation mechanism appears to be highly dependent on the structural compatibility of the amyloid precursor protein [241].

Secondary nucleation has been inferred for several proteins, including amyloid-β peptides [235,242], tau protein [243], α-synuclein [244,245], islet amyloid polypeptide (IAPP) [246], insulin [247], and bovine carbonic anhydrase [248].

Amyloid fibril formation may also be seeded by the presence of pre-formed aggregates. In this case, the primary nucleation event is negligible, leading directly to the growth phase, the absence of secondary mechanisms, and the polymerization process is expected to follow a single exponential function [249]. This is a consequence of the slower rate of primary nucleation when compared with the rate of addition of monomers onto an existing fibril (growth). This seeding process has been proposed to be an important factor in the propagation of the pathogenesis in most, if not all, amyloidoses [250,251,252,253,254,255,256].

### 5.2. Aggregation Via a Nucleation-Independent Mechanism

**Scheme 2:**
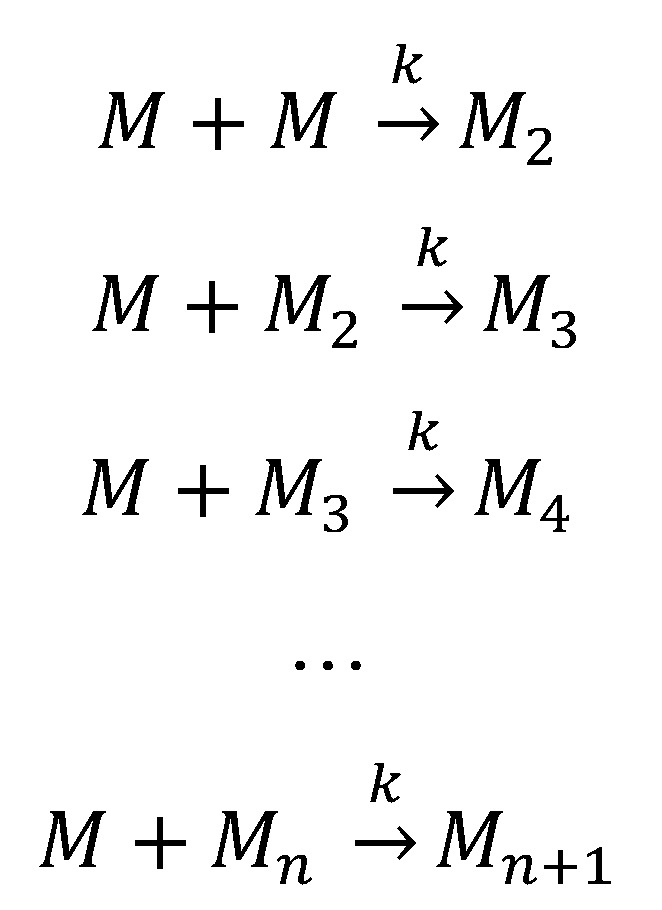
The sequential monomer (*M*) addition mechanism for protein aggregation with identical equilibrium constants (*k*) via a nucleation-independent pathway [257,258].

The nucleation-independent mechanism of protein aggregation (Figure 4 and Scheme 2) is an isodesmic or linear polymerization mechanism and may be exemplified by the simplest possible model for the formation of spherical oligomers or linear multimers [152]. This model is characterized by an infinite number of steps with identical rate constants (*k*) independent of the size of the aggregate (Scheme 2), resulting in an exponential polymerization curve with the absence of a lag phase. Once aggregation starts, the process undergoes downhill-polymerization. In this case, aggregation proceeds through a sequence of multiple energetically favorable steps, where the successive addition of amyloidogenic monomers to the growing aggregate is energetically favorable without the need of a multimeric nucleus. 

Generally, seeding does not increase the aggregation rate in a downhill-polymerization process. However, this model disregards other aggregation processes that can change the number, size and shape of oligomeric species. For these reasons, this model sometimes predicts incorrect length distributions of amyloid fibrils at equilibrium. Nevertheless, this kinetic model has been used to investigate the effect of mutations on the rate of amyloid fibril formation [259,260,261].

This aggregation mechanism was observed in transthyretin [262,263], among variants of the amyloid-β peptide [219,264,265], and also in the four-repeat domain of tau (Tau4RD) [266], β2-microglobulin [152], human and bovine serum albumins [267,268], HypF-N [269], FF domain [270], human muscle acylphosphatase [271], apolipoprotein C-II [272,273], and several SH3 domains [228,259,274,275,276].

### 5.3. The Energy Landscape View of Protein Aggregation

**Figure 5:**
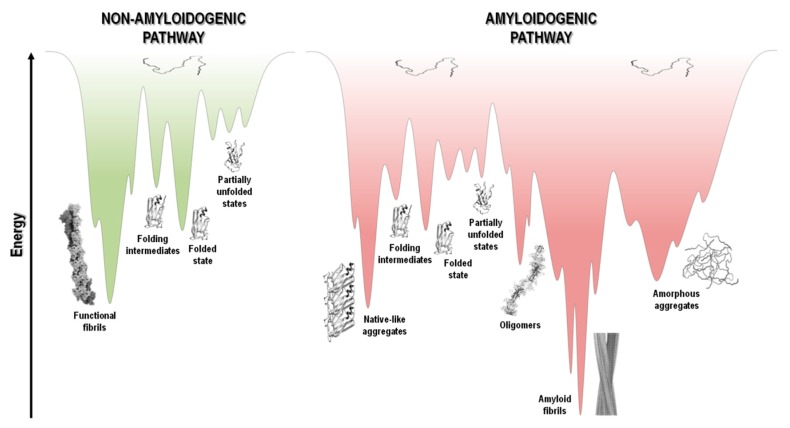
Schematic representation of funnel-shaped energy landscapes for protein folding (non-amyloidogenic pathway, green) and aggregation (amyloidogenic pathway, red). The surface exhibits the roughness of the protein energy landscape showing the possible conformational states adopted by the polypeptide chain. Unfolded, partially unfolded and folded species may be implicated in the aggregation landscape, as long as they are prone to establish intermolecular interactions and aggregate, thereby resulting in the formation of amorphous aggregates, amyloid fibrils, and native-like aggregates, respectively. Adapted from references [1,279,280].

The protein folding energy landscape available to each polypeptide chain includes a wide range of different conformational states and a multitude of pathways en route to the folded state. The energy landscape in the case of short polypeptide sequences tends to be a smooth funnel-shaped surface where the polypeptide chain folds quickly towards a single folded state [277]. On the other hand, larger proteins have rougher energy landscapes, with local minima and a population of intermediate states that eventually interconvert to the low energy folded state (Figure 5) [278,279].

At the top of the funnel, as depicted in Figure 5, the unfolded state of the polypeptide chain has high Gibbs free energy and high conformational entropy. Upon polypeptide chain folding, the number of conformational states and thus the conformational entropy decreases. Concurrently, the hydrophobic collapse and the increase in the number of intramolecular contacts leads to a decrease in free energy toward the native state occupying the global free energy minimum, yielding the necessary conformational stability of the folded state. However, changes in amino acid sequence, and/or chemical or biological environment, including changes in pH, temperature, ionic strength, pressure, agitation, shear forces, interaction with surfaces and many other factors, may tip the energetic balance towards a different free energy minimum. This is highlighted in Figure 5, where in parallel with the common folding funnel for a protein it is also depicted an aggregation funnel [280,281,282]. Regarding the protein aggregation process, the funnel-shaped free energy surface is potentially rougher and more complex, since the energy landscape encodes not only the relative stability of unfolded states, partially unfolded states, and folded states, but also the relative stability of amorphous aggregates, β-sheet-rich amyloid fibrils, and native-like aggregates (Figure 1 and Figure 5).

Protein aggregation involves several processes that are interconnected, such as folding, unfolding and partial folding/unfolding, conformational changes, formation of intermolecular interactions, and fibril nucleation, elongation and stationary phases (Figure 1 and Figure 4) [237,238,283,284,285]. There is well documented evidence that protein aggregation states may be formed not only by amyloidogenic intermediates but also by denatured and native states, with polypeptide chains establishing critical contacts with neighboring molecules through intermolecular interactions. Aggregated states are in general thermodynamically and kinetically favorable and it is a fine balance of forces that tip the processes towards a native soluble state or any type of aggregated state. It seems that most polypeptide chains under the right “stress” conditions tend to form extended β-sheet structures and thus amyloid aggregates [14,284,286,287]. The energy landscape of protein systems forming large aggregates is described by numerous peaks corresponding to different conformational states, which is the case of amyloids due to the heterogeneity of fibrillar morphology. Even under the same experimental conditions, a large number of polymorphic fibrils with distinct morphologies might be formed at the same time, emphasizing the complexity and multiplicity of the aggregation pathways [67,288]. The energy minimum of mature fibrils is deeper and sharper than the native state of a given protein (Figure 5), as suggested by the high stability of the fibrillar state [286,289,290].

Under specified conditions, a given polypeptide chain has its own folding and aggregation surface funnel. Each point on this surface expresses a specific and unique conformation of the protein. The profile of the energy landscape is affected by enthalpic contributions due to interactions between amino acids residues, and enthalpic and entropic contributions due to the interaction with the aqueous environment, as well as entropic contributions due to changes in conformational freedom of the polypeptide chain. The driving forces towards the low free energy state for both protein folding and aggregation are mainly hydrophobic in nature, with additional contributions from electrostatic and polar interactions, as well as hydrogen bonds [14,280,281,287]. In the case of amyloid fibrils, the cross-β motif conformation is stabilized essentially by polar interactions due to intermolecular hydrogen bonds, and intermediate aggregated species are formed by intermolecular hydrophobic and electrostatic interactions [284,291].

In the late 1990s [292] amyloid-like aggregates or fibrils were found to be formed in vitro under specific experimental conditions by proteins entirely unrelated to well-established amyloid diseases [293]. As we previously mentioned, under the right conditions, many if not most polypeptide chains may form amyloid. The term “amylome” was thus coined to describe all the proteins that can form amyloid-like fibrils [294]. There are authors that consider that the fibrillar amyloid state represents a standard state that every polypeptide chain can adopt under appropriate conditions, and that this state is the thermodynamic ground state. In this sense, the amyloid fibrillar conformation would be the universal global free-energy minimum of any polypeptide chain [295].

## 6. Conclusions

With the progresses being made in medical research and drug discovery, there is an optimistic view that protein misfolding diseases will become successfully diagnosed, prevented and treated. At the moment, the research into the “amyloid problem” is reaching a turning point, since the structures and properties of different amyloidogenic species involved in the amyloid cascade are finally being identified and, in some cases, structurally characterized. The improvement in resolution of molecular structures, with the contribution from X-ray, ssNMR and cryo-electron microscopy, has allowed a better understanding of how amyloid polymorphism may relate with different manifestations of amyloid diseases, and how different amyloid structures influence cellular function and may be influenced by tissue environment. This seems to be the perfect time to finally correlate protein aggregation mechanisms, structure of amyloid species, and how they impact the onset, severity and progression of different misfolding diseases. In turn, this is also the opportunity to set up therapeutic solutions based on rational approaches, and thus provide new hope to those many affected by amyloid diseases.

## Figures and Tables

**Table 1:** Human diseases associated with protein misfolding and amyloid aggregation [1,2].

Disease	Precursor Protein	Polypeptide Length (n° of Residues)	Structural Organization of Precursor
**Neurodegenerative Diseases**			
Alzheimer’s disease	Amyloid-β variants	37–44	IDP
Spongiform encephalopathies	Prion protein or its fragments	208	IDP and α-helical
Parkinson’s disease	α-synuclein	140	IDP
Frontotemporal dementia with Parkinsonism	Tau	352–441	IDP
Amyotrophic lateral sclerosis	Superoxide dismutase 1	153	β-sheet
Huntington’s disease	Huntingtin with polyQ expansion	3144	Mostly IDP
Neuroferritinopathy	Ferritin	175 or 183	α-helical
Familial British dementia	ABri	34	IDP
Familial Danish dementia	ADan	34	IDP
Familial amyloid polyneuropathy	Transthyretin variants	127	β-sheet
**Non-Neuropathic Systemic Amyloidosis**			
Amyloid light chain amyloidosis	Immunoglobulin light chains or its fragments	~90	β-sheet
Amyloid heavy chain amyloidosis	Immunoglobulin heavy chains or its fragments	~220	β-sheet
Amyloid A amyloidosis	Serum amyloid A protein fragments	45–104	α-helical and unknown fold
Familial Mediterranean fever	Serum amyloid A protein fragments	45–104	α-helical and unknown fold
Apolipoprotein A1 amyloidosis	Apo A-1 fragments	80–93	IDP
Senile systemic amyloidosis	Wild-type transthyretin	127	β-sheet
Familial amyloid cardiomyopathy	Transthyretin variants	127	β-sheet
Haemodialysis-related amyloidosis	β_2_-microglobulin	99	β-sheet
Lysozyme amyloidosis	Lysozyme variants	130	α-helical and β-sheet
Finnish hereditary amyloidosis	Fragments of gelsolin variants	53 or 71	IDP
**Non-Neuropathic Localized Amyloidosis**			
Type II diabetes	Islet amyloid polypeptide	37	IDP
Injection-localized amyloidosis	Insulin	21 and 30	α-helical
Gelatinous drop-like corneal dystrophy	Lactoferrin	691	α-helical and β-sheet
Medullary carcinoma of the thyroid	Calcitonin	32	IDP
Localized cutaneous amyloidosis	Galectin 7	136	β-sheet
Atrial amyloidosis	Atrial natriuretic factor	28	IDP
Cataracts	γ-crystallins	variable	β-sheet

IDP: Intrinsically disordered protein.

**Table 2:** Available online methods to predict amyloidogenic regions in amino acid sequences.

Method	Website	References
AGGRESCAN	http://bioinf.uab.es/aggrescan/	[70,71]
AGGRESCAN3D	http://biocomp.chem.uw.edu.pl/A3D/	[72,73]
AmyLoad	http://comprec-lin.iiar.pwr.edu.pl/amyload/	[74]
AmyloGram	http://www.smorfland.uni.wroc.pl/shiny/AmyloGram/	[75]
AmyloidMutants	http://amyloid.csail.mit.edu/	[76]
AMYLPRED	http://biophysics.biol.uoa.gr/AMYLPRED	[77]
AMYLPRED2	http://biophysics.biol.uoa.gr/AMYLPRED2	[78]
APPNN	http://cran.r-project.org/web/packages/appnn/index.html	[79]
BetaScan	http://groups.csail.mit.edu/cb/betascan/betascan.html	[80]
ClinVar	https://www.ncbi.nlm.nih.gov/clinvar/	[81]
Fibpredictor	http://nanohub.org/resources/fibpredictor	[82]
FISH Amyloid	http://www.comprec.pwr.wroc.pl/COMPREC_home_page.html	[83]
FoldAmyloid	http://bioinfo.protres.ru/fold-amyloid/oga.cgi	[84]
GAP	http://www.iitm.ac.in/bioinfo/GAP/	[85]
GOR V	http://gor.bb.iastate.edu/	[86,87]
MetAmyl	http://metamyl.genouest.org/	[88]
MILAMP	http://faculty.pieas.edu.pk/fayyaz/software.html#MILAMP	[89]
NetCSSP	http://cssp2.sookmyung.ac.kr/index.html	[90]
PASTA	http://protein.cribi.unipd.it/pasta/	[91,92]
PASTA 2.0	http://protein.bio.unipd.it/pasta2/	[93]
RFAmyloid	http://server.malab.cn/RFAmyloid/	[94]
SNPeffect 4.0	http://snpeffect.switchlab.org	[95]
STITCHER	http://stitcher.csail.mit.edu	[96]
TANGO	http://tango.crg.es/	[97]
Waltz-BD	http://waltzdb.switchlab.org	[98,99,100]
ZipperDB	http://services.mbi.ucla.edu/zipperdb/submit	[101]
Zyggregator	http://www-vendruscolo.ch.cam.ac.uk/zyggregator.php	[102]

**Table 3:** The pros and cons of the main methods that inform on amyloid structure at atomic level resolution.

Technique	Advances	Advantages	Disadvantages	References
X-ray and electron diffraction	1. Discovery of short protein segments that can themselves form amyloid fibrils and closely related crystals; 2. Development of synchrotron X-ray microbeams sufficiently focused and intense to determine a structure from a single crystal.	1. May yield atomic resolution; 2. Is not limited by the molecular weight of the specimen.	1. Well-ordered microcrystals needed; 2. The fibrils formed by some segments may represent the spines of polymorphs of full fibrils, but others may not; 3. The crystallized segment is only a few residues in length, thus nothing is revealed about the fibril structure outside the spine; 4. The steric zippers structures only show homo-steric zippers.	[23,60,62,126]
ssNMR	1. Innovations in high-field magnets, pulse sequences, high-resolution multi-channel magic-angle spinning (MAS) probes, ultrafast MAS, isotopic labeling schemes, use of quadrupolar nuclei as spectroscopic probes and solid-state dynamic nuclear polarization (DNP).	1. No need for crystals; 2. Structural information obtained on: identity of residues, recognition of parallel versus antiparallel β-sheets, register of strands within a sheet, and inter-residue contacts of amino acid side chains; 3. ssNMR-determined models show the overall conformation of the well-ordered portion of the chain around the protofilament spine; 4. Can be used to determine dihedral angles and inter-atom distances in the fibril subunits.	1. Amyloid-forming proteins are expressed recombinantly from media containing isotopically labeled amino acids; 2. Reliability of molecular models is highly dependent on the number of experimental constraints that have been collected; 3. The relative positions of atoms are not as accurately determined as in an atomic-resolution crystal structure; 4. The sensitivity of the experiments and spectral resolution decrease with the increase in molecular weight.	[127,129,130]
cryo-EM	1. Introduction of high-field microscopes; 2. New generation of direct detectors record the incident electrons in a thin, sensitive layer so that the signal is not scattered into surrounding pixels resulting in an improvement in image processing.	1. Near atomic-resolution structures of large molecular complexes without the need for crystals; 2. May yield the overall fibril structure: the number of protofilaments; the degree of twist; and, depending on the number of well-ordered specimens, information on the atomic structure of the fibril.	1. Due to a lack of contrast, images often have a very low signal-to-noise ratio, requiring highly advanced detection hardware and image processing; 2. Sample preparation can be difficult, not only to optimize thickness, but also to optimize particle distribution; 3. The most advanced cryo-EM equipment is very expensive.	[131,132,133]

**Table 4:** Early events that may lead to amyloid fibril formation.

**Nature of Monomeric Species**	**Description**	**References**
Native monomer	Normally folded proteins may retain a substantial tendency to aggregate through direct assembly of monomers in their native state when the native state exposes complementary surfaces.	[153,154,155,156]
Conformationally altered monomer	The native monomer has very low propensity to associate. Partial unfolding or conformational changes of the native monomer are required, resulting in a non-native species prone to aggregate.	[157,158,159,160,161,162]
Chemically modified monomer	Chemical modifications (deamidation, isomerization, hydrolysis, oxidation, photolysis, etc.) may cause conformational changes in native monomers, leading to species with high propensity to aggregate.	[163,164,165,166,167,168,169,170,171,172,173,174]
**Nature of Aggregation Interfaces**	**Description**	**References**
Gas-liquid interface	Hydrophobic–hydrophilic interfaces may induce aggregation reactions.	[175,176,177,178,179,180,181]
	Mechanical stress (agitation, stirring, pumping, or shaking) has been associated with cavitation which generates air bubbles and, consequently, the formation of an air-water interface which facilitates protein denaturation and aggregation.	[176,182,183,184,185,186,187,188,189]
	The use of beads during agitation accelerates the aggregation process by enhancing cavitation.	[190]
Solid-liquid interface	Solid-liquid interfaces may facilitate monomer encounters and initial monomer to monomer association and later further aggregation.	
	In vitro, interaction with glass, silicone, graphite, polypropylene, Teflon, mica, gold, etc. might lead to protein partial unfolding and aggregation.	[181,191,192,193,194]
	In vitro and in vivo, flow through tubes and vessels produce shear forces that may lead to protein partial unfolding and aggregation.	[195]
	Freeze-thaw cycles create new ice-water interfaces which may induce protein partial unfolding and aggregation.	[189,191,196]
	Presence of metal ions, in particular, Cu^2+^ and Zn^2+^, may promote aggregation of protein monomers bearing metal-ion binding sites or binding residues (e.g., histidines).	[197,198,199,200,201]
	Monomer association at the surface of biomembranes or biomolecules may also enhance aggregation.	[202,203,204,205,206,207,208,209,210]
